# Diagnosis of bovine rotavirus: an overview of currently available methods

**DOI:** 10.3389/fmicb.2025.1550601

**Published:** 2025-02-28

**Authors:** Ying Huang, Zhi Li, Yong Fu, Shu Qin Wang, Ming Kang, Ru Meng

**Affiliations:** ^1^Department of Veterinary Medicine, Qinghai University, Xining, China; ^2^Qinghai Academy of Animal Sciences and Veterinary Medicine, Qinghai University, Xining, China; ^3^Xining Animal Disease Control Center, Xining, China

**Keywords:** bovine rotavirus, diagnosis, etiological methods, serological methods, molecular biological methods

## Abstract

Bovine rotavirus (BRV) is a significant pathogen that causes diarrhea in calves, profoundly impacting the cattle industry and resulting in substantial economic losses. Currently, the established diagnostic approaches for BRV primarily include etiological methods, such as electron microscopy, virus isolation, and culture; serological methods, including enzyme-linked immunosorbent assay (ELISA), latex agglutination test (LAT), and immunofluorescence techniques; and molecular biological methods, such as reverse transcription-polymerase chain reaction (RT-PCR), real-time quantitative PCR (qPCR), and loop-mediated isothermal amplification (LAMP), as well as next-generation sequencing (NGS) technology. This review summarizes the current diagnostic methods for BRV, discusses their advantages and disadvantages, and presents future perspectives on BRV diagnosis, aiming to provide valuable references for the effective diagnosis and control of BRV-related diseases.

## 1 Introduction

Rotavirus is a zoonotic pathogen recognized as the predominant cause of severe diarrhea in children under the age of five, and in various young animal populations globally (Reactions Weekly, 2024). Additionally, rotavirus frequently serves as a contributing factor to, or a complication of, other diarrheal diseases (Alkoshi et al., 2014), thereby significantly affecting both human health and animal husbandry and garnering considerable attention from the scientific community. World Health Organization (2021) advocated the inclusion of rotavirus vaccines in all national immunization programs, prioritizing them.

Bovine rotavirus disease is an acute gastrointestinal infectious condition that significantly affects the global cattle industry, leading to substantial economic losses. The causative agent of this disease, bovine rotavirus (BRV), is classified within the Reoviridae family. This pathogen predominantly impacts calves, manifesting symptoms such as vomiting, diarrhea, and dehydration (Miyabe et al., 2020; Barsoum, 2022). The primary pathological findings associated with BRV infection include the presence of curd and milk in the stomachs of young animals, thin and translucent walls of the small intestine, liquid intestinal contents that are gray–yellow or gray–black in color, enlarged mesenteric lymph nodes, and atrophy and shortening of the small intestinal villi. BRV is transmitted primarily via the fecal-oral route and is located predominantly within the intestinal tract. Following extensive replication in the intestines, the virus is excreted in feces, leading to contamination of drinking water, feed, utensils, and soil, which can subsequently infect healthy cattle through the digestive system upon contact (Santiana et al., 2018). Some research has suggested that BRV may also be transmitted through small-particle aerosols in the air; however, there is currently no direct evidence to substantiate this hypothesis (Dennehy, 2000). Rotavirus was discovered in 1973, when Australian researcher Bishop identified it in ultrathin sections of the duodenum of patients suffering from gastroenteritis, thereby establishing its association with the disease (Bishop et al., 1973).

BRV is classified as an RNA virus characterized by the absence of an envelope, icosahedral symmetry, a diameter of approximately 70 nm for the complete viral particle, and a double-layered capsid. Its nomenclature is derived from its resemblance to a wheel (Männikkö, 2011; Yan et al., 2024). The cultivation and replication of this virus in cell culture present significant challenges, as proliferation is typically restricted to certain animal strains, and the cytopathic effects observed during the replication process are relatively mild. Notably, the rotavirus responsible for diarrhea in neonatal calves can replicate in MA-104 cells derived from rhesus monkey fetal kidneys, resulting in pronounced lesions (Bohl et al., 1984). The rotavirus genome consists of 11 segments of double-stranded RNA, which encode six structural proteins (VP1–VP4, VP6, and VP7) and six nonstructural proteins (NSP1–NSP6) (Vlasova et al., 2017). The VP6 protein serves as the inner capsid protein of rotavirus and functions as the group-specific antigen across various animal species and humans. It is recognized as the most conserved sequence among the 11 rotavirus gene segments, making it a preferred target for detection studies (Possatti et al., 2016). The outer capsid proteins of the rotavirus particle, VP7 and VP4, are responsible for inducing neutralizing antibodies and determining the G and P genotypes of the virus, respectively, and are thus extensively utilized in vaccine research. Currently, 42 G genotypes and 58 P genotypes of group A rotavirus have been identified (Cheng et al., 2021; Aksoy and Azkur, 2023). Given that the viral genome consists of multiple segments, coinfection of cells by viruses from different sources can lead to reassortment, particularly among viruses within the same serogroup. This genetic recombination has the potential to give rise to novel viral strains.

**Figure 1:**
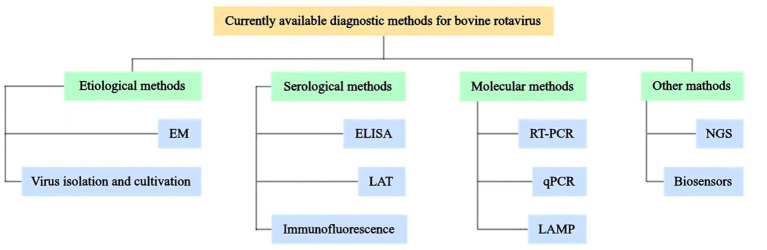
Schematic representation of currently available diagnostic methods for BRV. Various diagnostic methods available for BRV have been established, which include etiological methods, such as electron microscopy observation (EM) and virus isolation and cultivation; serological methods, such as enzyme-linked immunosorbent assay (ELISA), latex agglutination test (LAT), and immunofluorescence techniques; molecular methods, such as reverse transcription-polymerase chain reaction (RT-PCR), real-time quantitative PCR (qPCR), and loop-mediated isothermal amplification (LAMP); other methods, such as next-generation sequencing (NGS) and biosensors.

Bovine rotavirus disease represents a significant infectious threat to animal health and has considerable repercussions for the cattle industry. The prompt and precise diagnosis of BRV infection is crucial. To date, a variety of diagnostic techniques have been developed, encompassing etiological, serological, and molecular biological methods for the identification of BRV. This article offers a thorough examination of the different detection methods for BRV, highlighting their respective characteristics, advantages, and limitations (Table 1), with the objective of providing essential support for the diagnosis, prevention, and management of BRV (see Figure 1).

**Table 1:** Comparison of currently available diagnostic methods for BRV.

Test	Sensitivity	Specificity	Positive rate	Sample source in the literature	References
EM	22/28 (79%) compared to ELISAs and LATs	100% compared to ELISAs and LATs	No data	Fecal samples	de Beer et al. (1997)
TEM	No data	No data	No data	MA-104 cell suspensions infected with BRV DQ2020	Li et al. (2024)
Virus isolation and cultivation	No data	No data	2/20 (10%)	Fecal samples	Ates and Yesilbag (2023)
Direct sandwich ELISA	No data	No data	17/400 (4.3%)	Fecal samples	Abdou et al. (2021)
Indirect ELISA	21/24 (87.5%) compared to PCR	100% compared to PCR	21/24 (87.5%)	Fecal samples	Niu et al. (2024)
LAT	26/62 (42%) compared to EM	No data	45/375 (12%)	Fecal samples	Sukura and Neuvonen (1990)
	100% compared to ELISA	26/27 (96.3%) compared to ELISA	No data	Fecal samples	Al-Yousif et al. (2001)
Immunofluorescence techniques	No data	No data	38/100 (38%)	Intestinal membrane	Mitov et al. (1984)
RT-PCR	No data	No data	45/200 (22.5%)	Fecal samples	Uddin Ahmed et al. (2022)
End-point multiplex PCR/RT-PCR	10 IU	No data	16/35 (45.7%)	Fecal samples	Pedroso et al. (2023)
RT-qPCR	No data	No data	475/833 (57%)	Fecal samples	Castells et al. (2020)
Triplex LAMP-LFD	2.43 × 10^1^ copies/μL	155/156 (99.3%) compared to qPCR	No data	Anal swab samples	Xu et al. (2024)
NGS	No data	No data	No data	Fecal samples	Minami-Fukuda et al. (2013)
Biosensor	5 copies/mL	2.14 × 10^2^ copies/mL	No data	No data	Cho et al. (2022)

## 2 Etiological methods for the diagnosis of BRV

### 2.1 Electron microscopy observation

Under electron microscopy, BRV exhibits a characteristic “wheel” morphology. Currently, two primary techniques of electron microscopy are employed: direct electron microscopy and immunoelectron microscopy. Chasey and Labram (1983) observed fecal samples from naturally infected cattle using electron microscopy and identified three distinct assemblies with regular structures, which they designated rotatube 1, rotatube 2, and rotatube 3. Subsequent analysis revealed that these assemblies were composed of the capsid proteins of rotavirus, thereby establishing their association with the virus. However, this study also has certain limitations. The analysis was solely conducted via electron microscopic observation, lacking direct evidence of the components of these assemblies. de Beer et al. (1997) evaluated the efficacies of three detection methods, electron microscopy, enzyme-linked immunosorbent assay (ELISA), and latex agglutination test (LAT), for the identification of bovine rotavirus in the feces of young calves exhibiting diarrhea. In this study, electron microscopy was utilized as the gold standard and was compared with polyacrylamide gel electrophoresis (PAGE), underscoring the significance of electron microscopy in the detection of BRV and its distinctions from other diagnostic techniques. Although electron microscopy serves as a unique method for the direct observation of viral particles, its application in large-scale detection is hindered by certain limitations. Li et al. (2024) identified BRV-like particles, approximately 80 nm in diameter, in MA-104 cell suspensions infected with the BRV DQ2020 strain through transmission electron microscopy (TEM) But electron microscopic observation is merely employed to confirm that this strain possesses typical morphological characteristics of rotavirus and cannot serve as a direct identification basis. Despite the advantages of directness, accuracy, and rapidity associated with electron microscopy, its high operational costs, complex procedures, and there are other viruses that can have similar capsid organization and there is no way to definitively confirm it is a rotavirus in theory. Moreover, electron microscopy can suffer from poorer analytical sensitivity and throughput, thereby limiting its capacity to fulfill the demands for rapid diagnostic capabilities. Electron microscopic observation was once a crucial method for the diagnosis of bovine rotavirus and was frequently employed in the early stage of virus discovery. With the advancement of technology, its application frequency has declined in the proportion of overall diagnostic methods, yet it is still applied in specific circumstances. In scientific research endeavors such as the study of the morphological variations of the virus and the discovery of new strains, electron microscopic observation still holds an irreplaceable position. It can visually display the morphological changes of the virus and furnish an important basis for the study of virus evolution and variation.

### 2.2 Virus isolation and cultivation

Virus isolation and cultivation represent the most straightforward and effective methodologies for pathogen identification. Currently, the cell lines used for the isolation of BRV include MA-104 (rhesus monkey fetal kidney cells), CaCo-2 (human colon adenocarcinoma cells), ST (porcine testicular cells), and Marc-145 (African green monkey embryonic kidney cells). Notably, MA-104 cells are sensitive to BRV, allowing for viral replication and the manifestation of cytopathic effects. In practice, diarrheal fecal samples are inoculated into MA-104 cells, which are then subjected to continuous subculture and microscopically monitored daily until cytopathic changes are observed. Typically, after several passages, distinct and regular cytopathic effects can be identified. Using primary fetal bovine kidney cells, Theodoridis et al. (1979) successfully isolated and cultured BRV from the diarrheal feces of calves and observed cytopathic effects indicative of BRV infection. Similarly, Castells et al. (2018) isolated rotavirus A (RVA) from five out of 10 cell cultures derived from water samples collected from dairy farms, confirming the activity of the detected RVA particles. Elkady et al. (2021) inoculated treated fecal samples into MA-104 cells, observed characteristic cytopathic effects, and successfully isolated both group A and group B BRVs from fecal samples of Chinese calves. Ates and Yesilbag (2023) also inoculated treated fecal samples into the MA-104 cell line and reported that 2 out of 20 samples produced cytopathic effects, leading to the successful isolation of BRV. However, these scholars were not compared to a gold standard method. Isolation and culture can only detect infectious live viruses and cannot detect viral nucleic acids or protein fragments that are non-infectious yet still present in the samples. In some cases, the virus may be inactivated and unable to grow after isolation and culture, but its presence can still be detected through nucleic acid testing or immunoassay, which is crucial for comprehending the transmission and infection range of the virus. At the same time, it typically takes several days or even weeks from sample inoculation to the observation of obvious CPE. For situations where rapid diagnosis results are urgently required to adopt prevention and control measures, isolation and culture cannot meet the demand for rapid diagnosis, cannot provide a basis for disease prevention and control promptly during an outbreak, and hinder the provision of real-time results for clinical cases. Consequently, the clinical applicability of these methods is limited.

## 3 Serological methods for the diagnosis of BRV

### 3.1 Enzyme-linked immunosorbent assay

The enzyme-linked immunosorbent assay (ELISA) is a diagnostic technique predicated on the specific interaction between antigens and antibodies, with results quantified through the measurement of sample absorbance. Presently, various ELISA methodologies exist, including direct, indirect, double-antibody sandwich, and competitive approaches. Kharalambiev et al. (1983) developed an ELISA detection method utilizing the double-antibody sandwich principle for the identification of BRV antigens in fecal samples, facilitating rapid rotavirus diagnosis and offering a dependable means for detecting BRV infections. Bertoni et al. (2021) employed polyclonal indirect ELISA (iELISA) (Garaicoechea et al., 2006; Badaracco et al., 2013) to detect RVA antigens in fecal samples and utilized double-antibody sandwich ELISA (Fernandez et al., 1996; Parreno et al., 2004) to determine the IgG1 antibody titer for RVA. These findings indicated that various rearing practices significantly influence BRV infection rates in newborn calves during the initial 2 months of life, thereby providing valuable insights for cattle farms in selecting appropriate calf rearing methods. However, in this study, only ELISA was employed to detect the viral antigens and antibodies in fecal and serum samples, which might lead to false positive or false negative results. Moreover, for calves with low-level infections or in the early stage of infection, ELISA may fail to detect the viral antigens, resulting in missed detections and influencing the judgment of the true situation of viral infection. Abdou et al. (2021) conducted a prevalence study of RVA in 400 cattle fecal samples using an immunochromatographic assay (IC) and direct sandwich ELISA, revealing prevalences of 5.3% via IC and 4.3% via ELISA. This study demonstrated that IC is more sensitive than ELISA for detecting RVA antigens in fecal samples. Seid et al. (2020) performed BRV screening on 83 fecal samples from diarrheal calves under 4 weeks of age, alongside 162 nondiarrheal samples. Among the diarrheal samples, 6 (7.2%) tested positive for BRV antigens by Ag-ELISA, whereas all nondiarrheal samples tested negative. Tatte et al. (2019) collected 300 fecal samples from both diarrheic and healthy animals on farms in western India and detected RVA antigens in 3.1 to 25% of cattle using ELISA. Debelo et al. (2021) analyzed fecal samples from 110 calves aged 30 days or younger across 57 dairy herds in Addis Ababa, Ethiopia, using sandwich ELISA. Among the 110 calves, 42 (38.18%) exhibited diarrheal symptoms during the study, with a BRV prevalence of 3.64% (4/110). Niu et al. (2024) expressed the BRV VP6 protein in a eukaryotic expression system, immunized BALB/c mice to obtain serum, and established an indirect ELISA for the BRV VP6 protein. This method is characterized by its specificity and high sensitivity, thereby providing technical support for effective BRV detection and epidemiological investigations. Given its high accuracy, sensitivity, rapidity, and capacity for high-throughput sample analysis under less stringent biosafety conditions, ELISA has become widely adopted in the clinical detection and epidemiological research of BRV. Nevertheless, ELISA also has certain limitations. If an epidemic breaks out on a farm and timely prevention and control measures need to be implemented, the detection speed of ELISA is difficult to meet the requirement. However, some rapid detection techniques, such as immunochromatographic test strips, are simple and quick to operate and can obtain test results within a short period, which are more suitable for on-site rapid detection. ELISA is mainly utilized to detect the antigen or antibody of the virus and can only determine whether there is a viral infection in the sample and the general situation of the infection but cannot conduct genotyping and variation analysis of the virus. However, nucleic acid sequencing technology can sequence the nucleic acid of the virus and precisely determine the genotype and variation sites of the virus, providing more detailed information for the research and prevention and control of the virus.

### 3.2 Latex agglutination test

The latex agglutination test (LAT) employs latex particles in conjunction with specific antibodies to detect the presence of antigens. In instances where bovine rotavirus (BRV) antigens are present in a sample, a latex agglutination reaction is initiated. Sukura and Neuvonen (1990) conducted a study involving 375 fecal samples obtained from 62 calves at various ages (1, 3, 5, 7, 10, and 20 days). Their findings revealed that 45 samples (12%) tested positive for BRV using LAT, whereas 10 samples (2.7%) were confirmed to be positive through electron microscopy (EM). Notably, all samples identified as positive by EM were also positive by LAT. Among the 62 calves, 26 (42%) were positive by LAT, and 8 (13%) were positive by EM. The study concluded that LAT was straightforward to perform and demonstrated greater sensitivity than did EM and thus may serve as a specific method for the detection of BRV infection. Al-Yousif et al. (2001) collected 63 fecal samples from diarrheic calves between November 1999 and May 2000, employing various detection methods, including LAT, the Rotazyme II enzyme-linked immunosorbent assay (ELISA), and the anti-rotavirus fluorescent antibody (FA) test following virus isolation. This evaluation aimed to assess the efficacy of the Virogen Rotatest latex agglutination test kit in identifying BRV antigens. The results indicated that LAT, as a rapid screening method, offers several advantages, including ease of operation, the absence of a requirement for costly equipment and specialized personnel, and a prolonged shelf life for reagents. But for samples with a low virus content, the detection sensitivity of this method is suboptimal, and there is a certain degree of false positive occurrence. Moosai et al. (1985) assessed a commercial latex agglutination test known as Rotalex (Orion Diagnostics, Finland) and compared its performance with those of four widely utilized laboratory detection methods: electron microscopy, immunofluorescence, polyacrylamide gel electrophoresis, and enzyme-linked immunosorbent assay. Their evaluation revealed that when Rotalex was conducted according to the manufacturer’s guidelines, it exhibited deficiencies in both specificity and sensitivity. However, following modifications, its performance aligned more closely with those of the other methods. In summary, although LAT is characterized by its simplicity and rapidity, making it suitable for onsite screening, its specificity and sensitivity are relatively low and warrant further enhancement. When the virus content in the sample is low, the agglutination phenomenon resulting from antigen-antibody binding may not be conspicuous, and false negative results are prone to occur. In the early stage of virus infection, the viral load in cattle is relatively small, and the latex agglutination test may not be capable of effectively detecting the presence of the virus, resulting in missed detections. However, nucleic acid detection techniques such as reverse transcription-polymerase chain reaction (RT-PCR) can significantly enhance the detection sensitivity through the amplification of viral nucleic acids and can effectively detect even when the virus content in the sample is extremely low, greatly reducing the risk of missed detections. And LAT generally can only provide qualitative or semi-quantitative results and cannot precisely determine the content of the virus in the sample. While real-time fluorescent quantitative PCR technology can not only detect the presence of the virus but also accurately determine the content of viral nucleic acids, providing more valuable data support for disease assessment and treatment decision-making.

### 3.3 Immunofluorescence techniques

Immunofluorescence techniques are categorized into two main types: direct and indirect immunofluorescence. Direct immunofluorescence involves the application of fluorescently labeled specific antibodies that bind directly to viral antigens present in fecal samples, which are subsequently examined using a fluorescence microscope. In contrast, indirect immunofluorescence entails the initial binding of unlabeled antibodies to viral antigens, followed by the application of fluorescently labeled secondary antibodies for detection purposes. Mitov et al. (1984) conducted a study on the intestinal mucosa of 100 diseased calves utilizing direct immunofluorescence, which revealed the presence of BRV antigens in 38.0% of the examined cases. Parwani et al. (1996) collected fecal samples from diarrheal adult dairy cows, inoculated the filtrates into sterile calves, and successfully identified BRV-B or antigens in intestinal epithelial cells through immunofluorescence staining, but this method may have cross-reactions, leading to false positive or false negative results. These findings underscore the high specificity and sensitivity of this methodology; however, the requirement for specialized fluorescence microscopy equipment limits its widespread adoption in veterinary clinics. And for samples with a low viral load, immunofluorescence detection may not be capable of effectively detecting viral antigens, prone to false negative results, resulting in missed detections and delays in the diagnosis and prevention and control of the disease. The interpretation of the test results has a certain degree of subjectivity. Especially when the fluorescence signal is weak or atypical, interpretation errors are prone to occur, affecting the accuracy and consistency of the diagnosis.

## 4 Molecular biological methods for the diagnosis of BRV

### 4.1 Reverse transcription-polymerase chain reaction

Reverse transcription-polymerase chain reaction (RT-PCR) is a molecular biology technique that effectively amplifies RNA fragments *in vitro* and is extensively utilized in the regulation of specific genes. Basera et al. (2010) conducted a study involving the collection of 128 fecal samples from diarrheal calves in northern India, where they identified BRV infection through RNA polyacrylamide gel electrophoresis (RNA-PAGE) and RT-PCR. They specifically targeted the group-specific VP6 gene in 13 samples (11.81%) that tested positive for BRV by RNA-PAGE, confirming that 10 of these samples were classified as BRV-A. Uddin Ahmed et al. (2022) collected 200 fecal samples from diarrheal calves across three regions in Bangladesh between January 2014 and October 2015, reporting a BRV positivity rate of approximately 23%. They further characterized the G and P genotypes of the BRV-positive samples through RT-PCR and sequencing, identifying G6P[11] (94.4%) and G10P[11] (5.6%) as the predominant genotypes. Ates and Yesilbag (2023) collected 20 fecal samples from diarrheal calves on a farm in Turkey and used RT-PCR to detect BRV nucleic acids in the cell supernatants of two samples (RV-36 and RV-38) that exhibited cytopathic effects (CPEs) in the MA-104 cell line and tested positive for rotavirus A (RVA) antigen by ELISA. RT-PCR targeting the VP6 gene region yielded a 379 bp amplicon for both positive samples, confirming the presence of BRV. Pedroso et al. (2023) developed an endpoint multiplex PCR/RT-PCR for diagnosing neonatal calf diarrhea (NCD). After optimizing the assay conditions and validating its specificity, sensitivity, and reproducibility, 95 samples were analyzed, of which 50 were positive for at least one target pathogen, with 35 representing single infections and 15 indicating mixed infections. Among the single infections, BRV was the most frequently detected pathogen (16/35). Nevertheless, in multiplex PCR reactions, the simultaneous presence of multiple primers may give rise to non-specific binding and generate non-specific amplification products, thereby interfering with the interpretation of the results and causing false positives. Especially when there are other microorganisms or nucleic acid fragments with similar nucleic acid sequences to the target pathogen in the sample, it is more prone to cause specificity issues. In summary, RT-PCR offers several advantages, including the requirement for minimal sample volume, high specificity, rapid execution, simplicity, and elevated sensitivity. This method is particularly advantageous for the screening of clinical diseases, as it can significantly reduce costs and expedite diagnosis. For cattle farmers, RT-PCR provides timely confirmation of herd health status, thereby minimizing unnecessary examinations and treatments and increasing the survival rates of affected cattle. However, impurities present in the sample, inhibitors, or differences in nucleic acid extraction methods may all impact the extraction efficiency of nucleic acids, resulting in insufficient or poor-quality viral nucleic acids extracted, thereby affecting the subsequent PCR amplification and leading to false negatives; while insufficient primer specificity and extremely trace amounts of nucleic acid contamination during the operation can give rise to false positive results.

### 4.2 Real-time quantitative PCR

Real-time fluorescence quantitative PCR (qPCR) integrates reverse transcription PCR (RT-PCR) with fluorescent dyes, enabling not only the detection of bovine rotavirus (BRV) infection but also the quantitative analysis of viral loads. Castells et al. (2020) conducted an analysis of 833 samples from dairy and beef calves in Uruguay using RT-qPCR and sequencing techniques and revealed that rotavirus A (RVA) was present in 57.0% of the samples. Notably, the detection rate in dairy calves (59.5%) was significantly greater than that in beef calves (28.4%). In a separate study, de Barros et al. (2018) collected 648 fecal samples from various animal species in northeastern Pará State, Brazil, between October 2014 and April 2016, targeting the NSP3 gene for RT-qPCR analysis of RVA. Their findings indicated that 27.5% (178/648) of the samples tested positive for RVA, with positive samples identified across multiple species, including birds, canids, bats, cattle, horses, small rodents, pigs, and felines; the positive rate in cattle was recorded at 14.6%. These findings suggest that RVA has the potential to disseminate among diverse animal populations, potentially facilitating cross-species transmission and genomic recombination. Furthermore, Benito et al. (2020) examined 237 fecal samples from diarrheal calves under 2 months of age in Spain and detected the presence of bovine group A rotavirus (RVA), *Cryptosporidium parvum*, and bovine coronavirus (BCoV) through RT-qPCR. Among these samples, 188 (79.3%) were positive for at least one pathogen, with 101 samples (42.6%) exhibiting mixed infections, and the RVA infection rate was determined to be 50.6%. Additionally, Punia et al. (2023) developed and optimized a sensitive, specific, and reliable TaqMan probe-based RT-qPCR method for the rapid detection and quantification of enteric viruses in fecal samples. This method demonstrated amplification capabilities for RVA, bluetongue virus (BTV), and bovine coronavirus (BoCV) RNA that were 1,000 times more sensitive than those of traditional gel-based RT-PCR, exhibiting excellent repeatability and the ability to accurately quantify viral RNA loads in clinical samples. However, these scholars did not compare this method with the gold standard method. In summary, qPCR eliminates the need for electrophoresis, as the entire procedure is conducted in a closed-tube format, thereby minimizing the risk of false-positive results due to sample contamination. Compared with conventional RT-PCR, qPCR offers increased specificity and sensitivity, making it particularly suitable for the rapid detection of BRV. However, this method imposes more stringent requirements for primer design and experimental conditions, necessitating a higher level of technical expertise from operators (Ward et al., 2013). This indicates that this detection method, in comparison with other methods, has advantages as well as certain limitations. qPCR can only detect the nucleic acid of the virus and cannot determine whether the detected viral nucleic acid is from infectious viral particles or inactive viral fragments, etc., whereas virus isolation and culture can directly determine whether the virus could infect cells and reproduce. In the early stage of infection, the virus may not have replicated or released nucleic acids in large quantities. At this time, qPCR may not be able to detect viral nucleic acid, and there is a certain window period. Serological detection can shorten the detection window period to a certain extent by detecting early antibodies such as IgM and detect the infection earlier. qPCR has relatively high requirements for the quality of samples, while the colloidal gold immunochromatography method has relatively low requirements for the quality of samples. It can directly detect viral antigens in samples such as feces, and the processing of samples is relatively straightforward.

### 4.3 Loop-mediated isothermal amplification

Loop-mediated isothermal amplification (LAMP) employs specific primers and enzymes to facilitate nucleic acid amplification at a constant temperature. Xie et al. (2012) developed and optimized a reverse transcription loop-mediated isothermal amplification (RT-LAMP) method for the rapid detection of BRV, which demonstrated good specificity and sensitivity. This method is anticipated to serve as a rapid and straightforward diagnostic tool for identifying BRV infections in calves. Additionally, Xu et al. (2024) introduced a triple loop-mediated isothermal amplification-lateral flow immunochromatographic dipstick (LAMP-LFD) detection method, which enables the simultaneous detection of three viruses: bovine viral diarrhea virus (BVDV), BRV, and bovine papillomavirus (BPV). This method was further evaluated using 156 anal swab samples, which yielded results that were consistent with over 99% of those obtained through quantitative polymerase chain reaction (qPCR). Given its high sensitivity and specificity, along with its independence from laboratory equipment or specific conditions, this detection method is poised for application in the rapid onsite identification of triple virus infections. Consequently, the LAMP reaction is characterized by its rapidity, lack of requirement for specialized instruments, and suitability for onsite detection. However, in comparison with qPCR, LAMP is challenging to provide accurate copy number of viral nucleic acids. In the event of an extremely low viral load, there may be a certain risk of missed detection. Moreover, LAMP is relatively more influenced by inhibitors in the sample, such as impurities in feces, which may inhibit the reaction and reduce the reliability of the detection. Serological methods such as ELISA and immunofluorescence can directly detect the antigen of the virus and can be employed to analyze information such as the antigenic characteristics and serotype of the virus, which is beneficial to understanding the immunogenicity and epidemiological characteristics of the virus. And LAMP cannot detect the antigenic characteristics of the virus. Compared with gene sequencing, LAMP cannot obtain complete gene information and has a narrower detection range.

## 5 Other methods for the diagnosis of BRV

### 5.1 Next-generation sequencing

Next-generation sequencing (NGS) technology is mainly founded on the sequencing analysis of viral nucleic acids. Firstly, viral nucleic acids are extracted from the samples infected with bovine rotavirus. The cDNA after reverse transcription is fragmented by techniques such as PCR to generate short fragments suitable for sequencing. Then, specific adapters are added to these fragments to construct a sequencing library. Subsequently, the DNA fragments in the library are loaded onto the sequencing platform. Through different sequencing technologies and in accordance with the principle of base complementary pairing, the base sequence of each fragment is determined successively. Finally, a considerable amount of short sequence data obtained by sequencing is compared, spliced, and analyzed with the known genomic sequences of bovine rotavirus with the assistance of bioinformatics software. Based on information such as sequence similarity and coverage, it is determined whether bovine rotavirus exists in the samples and its genotype, genetic variation, etc. are ascertained, thereby achieving an accurate diagnosis of bovine rotavirus. NGS enables comprehensive sequencing of all nucleic acids present in a sample, facilitating the detection of BRV while concurrently analyzing other pathogens. This capability enhances the understanding of the disease’s etiology. In a study conducted by Minami-Fukuda et al. (2013), the sensitivities of human rotavirus rapid antigen detection (RAD) kits, reverse transcription-polymerase chain reaction (RT-PCR), and next-generation sequencing (NGS) for identifying BRV-A were evaluated. NGS was applied to 13 fecal samples that tested negative by RT-PCR, yielding reads from all samples, with two samples encompassing all 11 genomic segments. This finding underscores the sensitivity of NGS and its utility in analyses that are less reliant on specific primers and genotype screening. Additionally, Dennis et al. (2014) utilized NGS to characterize a novel Ghanaian human-bovine reassortant rotavirus strain of the G8P[6] type, which has significant implications for understanding the evolution and interspecies transmission of BRV. Furthermore, Li et al. (2024) employed Illumina next-generation sequencing (NGS) to sequence the complementary DNA (cDNA) derived from the supernatant of a cell culture medium and successfully obtained the complete genome of the BRV strain DQ2020. However, NGS technology may produce false negative results. For instance, failure to collect adequate viruses during the sample collection process, improper sample preservation and transportation leading to the degradation of viral nucleic acids, and unreasonable parameter settings during the bioinformatics analysis process may all influence the recognition and judgment of viral sequences. Nonetheless, the complexity and high cost associated with NGS limit its application primarily to scientific research and extensive epidemiological surveillance.

### 5.2 Biosensors

Biosensors fix the specific antibody or nucleic acid aptamer against bovine rotavirus on the sensor. When the sample containing bovine rotavirus encounters the sensor, the virus antigen or aptamer specifically binds to the virus, inducing a change in the sensor signal, thereby achieving qualitative or quantitative detection of bovine rotavirus. Biosensors have high specificity, can precisely identify bovine rotavirus, reduce cross-reactions with other pathogens, and meanwhile can achieve quantitative analysis of bovine rotavirus, accurately determine the virus content, which is conducive to assessing the severity of the disease and the infection process. Moreover, some biosensors are small, convenient to carry and operate, and can be used for on-site detection without sending samples to professional laboratories. They can detect epidemics in a timely manner and adopt prevention and control measures. Cho et al. (2022) introduced an electrochemical biosensor utilizing affinity peptides for the rapid detection of BRV. This system demonstrated low limits of detection and quantification, suggesting its potential as an effective sensor platform for monitoring BRV and investigating novel detection methodologies for this virus. However, the preparation process of biosensors frequently involves intricate techniques and procedures, rendering their production costs high and restricting large-scale applications. Currently, biosensors for bovine rotavirus might not fully attain the level of traditional gold standard detection methods in terms of detection accuracy, with certain false positive or false negative rates, influencing the reliability of the diagnosis.

## 6 Conclusions and perspectives

BRV can be identified through various diagnostic methodologies. Traditional techniques such as electron microscopy and virus isolation and culture serve as foundational approaches for BRV diagnosis. Electron microscopy allows for direct visualization of viral morphology; however, it requires high sample concentrations and sophisticated equipment. Conversely, virus isolation and culture are regarded as the gold standard for diagnosis, although they are characterized by time- and labor-intensive processes. Serological methods, including enzyme-linked immunosorbent assay (ELISA), lateral flow assays (LAT), and immunofluorescence techniques, offer relatively straightforward operational procedures for detecting antibodies or antigens. Nonetheless, these methods may exhibit limitations in terms of sensitivity and specificity. Molecular biological techniques, such as reverse transcription-polymerase chain reaction (RT-PCR), quantitative PCR (qPCR), and loop-mediated isothermal amplification (LAMP), demonstrate enhanced specificity and sensitivity. Notably, LAMP facilitates rapid and efficient detection; however, it is susceptible to false-positive results. Additionally, next-generation sequencing (NGS) provides extensive genomic insights, aiding in the investigation of viral evolution and epidemiology, and holds potential for application in BRV diagnosis.

The precise diagnosis of BRV is essential for the effective management of neonatal calf diarrhea (NCD) and for the surveillance of viral variations. Despite the availability of several diagnostic approaches for BRV, considerable variability in their sensitivity and specificity and certain inherent limitations exist. Future efforts should focus on the optimization of these diagnostic techniques to increase their accuracy, sensitivity, and specificity. Additionally, there is a pressing need to develop more rapid, precise, and specific diagnostic methodologies, such as next-generation sequencing (NGS), which can yield comprehensive genomic information regarding BRV strains and facilitate a deeper understanding of viral evolution and epidemiology.

Subsequently, Sudan Guray et al. (2021) introduced an innovative, rapid, and pen-sided diagnostic test for the detection of bluetongue virus (BTV) utilizing a multiwalled carbon nanotube (MWCNT)-based immunosensor. This development serves as a prototype for the creation of straightforward and cost-effective diagnostic tools. Sahoo et al. (2023) engineered a lateral flow device (LFD) employing secondary antibody-derived gold nanoprobes for the swift and sensitive identification of bluetongue (BT). Compared with indirect ELISA, this device demonstrates enhanced sensitivity and specificity, facilitating prompt and precise onsite diagnosis of BT. Given that both BTV and BRV are members of the Reoviridae family and share fundamental characteristics, the MWCNT-based immunosensor and LFD utilizing secondary antibody-derived gold nanoprobes may also be relevant for the detection of BRV. Lin et al. (2023) provided a comprehensive overview of the detection methodologies for SARS-CoV-2 using microfluidic technology. They highlighted the integration of a microfluidic digital chip with CRISPR/Cas12a-assisted RT-PCR. Furthermore, the microfluidic device is designed to interface with smartphones, enabling the reporting and tracking of test results while facilitating rapid detection and high-throughput analysis. This presents new avenues and references for the advancement of diagnostic methodologies for BRV.

The existing multiplex PCRs employed for the detection of BRV primarily target the virus itself, along with one or more additional pathogens associated with bovine diarrhea. Future advancements may involve the development of multiplex PCR assays capable of identifying various serogroups (particularly groups A and B), as well as recombinant genotypes of BRV.

In summary, forthcoming investigations into BRV should prioritize the examination of viral variations, the ongoing enhancement of diagnostic methodologies, the establishment of more efficient strategies for the prevention and management of BRV, and the advancement of the overall health of the cattle industry.
